# Impact of Intrahost NS5 Nucleotide Variations on Dengue Virus Replication

**DOI:** 10.3389/fmicb.2022.894200

**Published:** 2022-07-05

**Authors:** Dayna Cheng, Sheng-Wen Huang, Wei-Xin Chin, Su-Jhen Hung, Huey-Pin Tsai, Justin Jang Hann Chu, Chiao-Hsuan Chao, Jen-Ren Wang

**Affiliations:** ^1^Institute of Basic Medical Sciences, College of Medicine, National Cheng Kung University, Tainan, Taiwan; ^2^Department of Medical Laboratory Science and Biotechnology, College of Medicine, National Cheng Kung University, Tainan, Taiwan; ^3^National Mosquito-Borne Diseases Control Research Center, National Health Research Institutes, Tainan, Taiwan; ^4^Department of Microbiology and Immunology, Infectious Diseases Translational Research Program, Yong Loo Lin School of Medicine, National University of Singapore, Singapore, Singapore; ^5^Department of Pathology, National Cheng Kung University Hospital, Tainan, Taiwan; ^6^National Institute of Infectious Diseases and Vaccinology, National Health Research Institutes, Tainan, Taiwan

**Keywords:** dengue virus, quasispecies, virulence, next-generation sequencing, intrahost variations on dengue replication, disease severity

## Abstract

Due to the nature of RNA viruses, their high mutation rates produce a population of closely related but genetically diverse viruses, termed quasispecies. To determine the role of quasispecies in DENV disease severity, 22 isolates (10 from mild cases, 12 from fatal cases) were obtained, amplified, and sequenced with Next Generation Sequencing using the Illumina MiSeq platform. Using variation calling, unique wildtype nucleotide positions were selected and analyzed for variant nucleotides between mild and fatal cases. The analysis of variant nucleotides between mild and fatal cases showed 6 positions with a significant difference of *p* < 0.05 with 1 position in the structural region, and 5 positions in the non-structural (NS) regions. All variations were found to have a higher percentage in fatal cases. To further investigate the genetic changes that affect the virus’s properties, reverse genetics (rg) viruses containing substitutions with the variations were generated and viral growth properties were examined. We found that the virus variant rgNS5-T7812G (G81G) had higher replication rates in both Baby hamster kidney cells (BHK-21) and Vero cells while rgNS5-C9420A (A617A) had a higher replication rate only in BHK-21 cells compared to wildtype virus. Both variants were considered temperature sensitive whereby the viral titers of the variants were relatively lower at 39°C, but was higher at 35 and 37°C. Additionally, the variants were thermally stable compared to wildtype at temperatures of 29, 37, and 39°C. In conclusion, viral quasispecies found in isolates from the 2015 DENV epidemic, resulted in variations with significant difference between mild and fatal cases. These variations, NS5-T7812G (G81G) and NS5-C9420A (A617A), affect viral properties which may play a role in the virulence of DENV.

## Introduction

Dengue virus (DENV) is a positive-sense single stranded RNA (+ssRNA) that belongs to the *Flaviviridae* family and have affected millions of individuals world-wide. Symptoms of flavivirus infection can range anywhere from mild fever and malaise to fatal encephalitis and hemorrhagic fever (Mukhopadhyay et al., 2005; Greaves and Hunt, 2010; Burrel et al., 2017). Clinical manifestations of DENV are classified into two categories: without and with warning signs. In severe cases, thrombocytopenia and plasma leakage may be observed which can lead to hemorrhage (dengue hemorrhagic fever, DHF) and shock (dengue shock syndrome, DSS; Gubler Duane, 1998; Hadinegoro, 2012; Bäck and Lundkvist, 2013; Wan et al., 2013; Megawati et al., 2017). Dengue fever (DF) is observed most frequently as compared to DHF and DSS. In Taiwan, in 1915, 1931, and 1942 there were DF outbreaks island wide (Koizumi et al., 1918; Chen, 2018). Other outbreaks occurred in 1981, 1987, 1988, 2001, 2002, and 2007 (Hsieh et al., 1982; Wu, 1986; Ko, 1989; Chen et al., 2008; Wang et al., 2016b). The 2002 outbreak was similar to that of 1987–1988 whereby the numbers of indigenous dengue cases were approximately 5,000 and 4,000, respectively (Chen et al., 2008; Chen, 2018). Taiwan has experienced many outbreaks at different levels; however, for the first time in the history of Taiwan, the island was faced with two consecutive outbreaks in 2014 and 2015. The 2015 outbreak was by far the greatest and most severe dengue outbreak Taiwan had ever encountered, resulting in over 43,000 dengue cases, including 228 deaths in Southern Taiwan (Wang et al., 2016a; Chang et al., 2018; CDC, R.O.T.C., 2022). In Tainan City alone, 22,741 cases with 112 deaths were reported (CDC, Taiwan). During this outbreak, a larger number of elderlies had been reported to be infected with the virus as compared to the other age brackets. This was also the year where the highest number of fatal cases from DENV was recorded. Apart from symptoms displayed by patients, the differences in the genetic make-up of the virus between mild and fatal cases are still unknown.

Dengue virus, like many other RNA viruses, tend to have a high mutation rate. This is due to the low fidelity of the RNA-dependent RNA polymerase (RdRp) which is present in these viruses (Domingo, 1997; Yu et al., 2007; Van Slyke et al., 2012). The average rate of mutation for RNA viruses is one mutation per genome per cycle (Vignuzzi et al., 2006). Due to this high mutation rate, rather than all the viruses replicated being of a single haplotype containing the same sequence, the sequences within viral population that is produced would differ from the parental strain. This phenomenon has been termed as “quasispecies” (Domingo et al., 2021). Quasispecies has been described as a cloud of diverse variants that are genetically linked through mutations, interact cooperatively on a functional level, and contributes to the characteristics of the population together (Lauring and Andino, 2010). The quasispecies theory developed by Eigfren and Schuster is defined as a mathematical framework that was initially formulated to explain the evolution of life in the “precellular RNA world” (Eigen, 1971; Eigen and Schuster, 1978; Vignuzzi et al., 2006; Lauring and Andino, 2010). It is also believed that quasispecies play an important role in the survival and evolution of RNA viruses as well as in the pathogenesis of disease.

Vignuzzi et al. (2006) had found that quasispecies is essential for adapting to and surviving new selective pressures in changing environments, and that the cooperation between viral variants allows for successful colonization of the ecosystem. In that study they provided evidence that there is a link between mutation rate, population dynamics, and pathogenesis (Vignuzzi et al., 2006). Huang et al. (2017) had focused on enterovirus virus isolates collected from different tissues from a single patient. It was determined that despite the isolates being from a single patient, the viral population from one tissue differed from that of another (Huang et al., 2017). This thus shows how quasispecies played a role in surviving new selective pressures and bottlenecks. The importance of quasispecies in viral infections have also been reported for human immunodeficiency virus (HIV), hepatitis viruses, and the newly pandemic SARS-CoV-2 virus (Farci et al., 2000; Herring et al., 2005; Fishman and Branch, 2009; McMichael et al., 2010; Hwang et al., 2021; Sun et al., 2021). In a previous study, DENV had been found in plasma as a population of closely related genomes, and that this quasispecies was present *in vivo* (Wang et al., 2002). During a DENV-3 epidemic in Cuba, Rodriguez-Roche et al. (2016) had previously reported the change in the intrahost genetic diversity of a selected minor virus population becoming predominant toward the end of the epidemic (Rodriguez-Roche et al., 2016). More recently, Torres et al. had demonstrated the intrahost diversity in DENV-2 patients with different clinical outcomes and found that the *NS2B* gene was shown to be constrained. However, of the 124 non-synonymous intrahost single-nucleotide variants that were highly detected in more severe cases, no single variant was found to be associated with disease severity (Torres et al., 2021).

Next-generation sequencing (NGS), also known as high-throughput sequencing, is a massively parallel sequencing technology. There are several known sequencing technologies such as Ion Torrent, Pacific Biosciences (PacBio), and Illumina (Quail et al., 2012). All NGS platforms have the ability to sequencing of millions of small fragments of DNA in parallel. Bioinformatics analyses are used to piece these fragments together by mapping reads to a reference genome which are sequenced multiple times. Therefore, NGS provides high depth to deliver accurate data and an insight into unexpected DNA variation, and as the years progress, the technology used becomes faster, more sensitive, more accurate, and able to run even more data in a single sequencing run (Barzon et al., 2013; Behjati and Tarpey, 2013; Bahassi and Stambrook, 2014).

Using NGS, we hypothesized that the quasispecies of DENV-2 in the Taiwan 2015 epidemic played a role in disease severity between mild and fatal cases. This may also provide us with insight into potential DENV virulence determinants. In this study, we utilized next generation sequencing (NGS) and data analysis programs, to determine significant viral genomic variations between mild and fatal cases. We also generated reverse genetics (rg) systems for investigating whether the variants could show better replication and thermal stability compared to wildtype, thus indicating that the nucleotide variations obtained from quasispecies may impact viral properties and virulence of DENV.

## Materials and Methods

### Ethics Statement

This study was conducted in Tainan, Taiwan. Approval for the study was obtained from the Institutional Review Board (IRB) of National Cheng kung University Hospital (No. A-ER-106-133). The demographic and clinical information for the patients were de-linked prior to analysis, therefore, the informed consent was waived from patients prior to the study.

### Cells and Virus Isolates

C6/36 cells (ATCC: CRL-1660) maintained in RPMI-1640 (RPMI) with 10% fetal bovine serum (FBS) and 2% penicillin/streptomycin (P/S) at 29°C, and RPMI virus medium (2% FBS, 2% P/S) were used in virus isolation, virus culture, and amplification. Baby hamster kidney cells (BHK-21) and Vero cells were maintained in Eagle’s Minimum Essential Medium (EMEM) with 10% FBS, 2% P/S, and 1% sodium pyruvate at 37°C. BHK-21 cells (ATCC: CRL-12071) in EMEM virus medium (2% FBS, 2% P/S, 1% sodium pyruvate) were used in transfection and to analyze virus growth kinetics, temperature stability, and thermal sensitivity. Vero cells (ATCC: CCL-81), cultured in EMEM virus medium (2% FBS, 2% P/S, 1% sodium pyruvate) at 37°C, were used to analyze virus growth kinetics, temperature stability, thermal sensitivity and to obtain virus titers. Huh-7 cells maintained in DMEM containing 10% FBS and 2% P/S were used to analyze virus growth kinetics and thermal sensitivity.

Patient sera (Supplementary Table S1) from patients infected with dengue virus serotype 2 from the 2015 outbreak in Southern Taiwan were collected from the Virology Laboratory of National Cheng Kung University Hospital (NCKUH), Tainan, Taiwan. Viruses were isolated from clinical samples in C6/36 cells. Isolated viruses were further amplified and passaged for two more passages (P2) in C6/36 cells before RNA extraction for NGS analysis. Immunofluorescence staining was carried out to determine successful virus isolation and passaging using the primary antibody: mouse monoclonal antibodies to DENV 1–4 (GeneTex) and the secondary antibody: anti-mouse IgG conjugate FITC. The glass slides were observed under a fluorescent microscope.

### DENV-2 Gene Amplification and Next Generation Sequencing

Viral RNA was extracted from virus isolates (P2 viruses) using the QIAamp Viral RNA Mini Kit (QIAGEN). RNAs were reverse transcribed into cDNA using the SuperScript III Reverse Transcriptase (Invitrogen) or Maxima H Minus Reverse Transcriptase (ThermoScientific) and with anti-sense primer D2Rv3 and anti-sense primer D2R (Supplementary Table S2). The cDNAs were PCR amplified into two fragments using primer pairs: D2F with D2Rv3, and D2Fw4 with D2R, as previously designed (Chin-inmanu et al., 2012), using KOD plus enzyme (TOYOBO). The first fragment (D2F with D2Rv3) was amplified using the following PCR conditions: 95°C for 1 min, 65°C for 1 min, and 68°C for 6 min, for 30 cycles. The second fragment (D2Fw4 with D2R) was amplified using the following PCR conditions: 95°C for 1 min, 65°C for 1 min, and 68°C for 4 min, for 30 cycles. PCR products were pooled together, the fragments were purified by phenol/chloroform, and pellet was resuspended with 15 μl DEPC water. More than 1 μg of DNA was sequenced using the Illumina Miseq platform (Quail et al., 2012) by Genomics BioSci & Tech.

### Genetic Variation Analysis

DENV full length genome sequence for each isolate were assembled from dataset reads obtains from NGS *via De Novo* Assembly to produce a full-length consensus sequence. NGS dataset reads were aligned with the consensus sequence to produce haplotype construction data and variation frequency using QuasiRecomb (version 1.2; Töpfer et al., 2013). Genetic variation and variation frequency were analyzed from the QuasiRecomb data. Using another program, LoFreq (version 2.1.2; Wilm et al., 2012), unique positions with significance were determined and variations with value of ps < 0.05 (Chi-square) were selected for further analysis. The deep sequencing reads of DENV-2 were deposited in the NCBI Sequence Read Archive under the BioProject ID PRJNA814417.

### Haplotype Prediction and Analysis

Using QuasiRecomb, a quasispecies.fasta file was generated to produce a list of haplotype sequences and their percentage within the virus population of each sample. BioEdit Sequence Alignment Editor (version 7.2.5) was used to compare the sequences and extract the haplotype sequences. Each read within the file is considered to be a single haplotype and the percentage of that haplotype is stated. Using the number of haplotype sequences generated and the percentage of each haplotype, a pie chart is produced for visualization using Microsoft Excel. The number of haplotypes per sample was further used in comparing between mild and fatal cases (*t*-test).

### Construction of Infectious cDNA Clones

Site-directed mutagenesis was performed to construct infectious cDNA clones, containing various substitutions. Using a plasmid pDL-DENV2-EGFP-C60-10062016-A2 (pDENV2-1,006) as template (Lee et al., 2019), mutagenesis primers designed to contain the various substitutions were used. The pDENV2-1,006 plasmid contained modifications in the recombinant cassette whereby the duplicate capsid sequence that is located downstream of the EGFP gene only has the first 60 nucleotides (instead of 75) are modified with codon optimizations. The list of primers for the respective substitutions are found in Table S2. E-C1350A (T188T), NS5-T7812G (G81G), NS5-T8919G (C450W), NS5-C9420A (A617A), and NS5-C9938A (T790K) were amplified using the following PCR conditions: 95°C for 1 min, 65°C for 1 min, and 68°C for 4 min, for 30 cycles. NS3-A5990C (E490A) was amplified using the following PCR conditions: 95°C for 30 s, 65°C for 30 s, and 68°C for 1.5 min, for 30 cycles. The amplicons were pooled and purified using Geneaid Gel Extraction Kit (Cat. No. DF300). The respective fragments of E-C1350A (T188T), NS5-T7812G (G81G), NS5-T8919G (C450W), NS5-C9420A (A617A), and NS5-C9938A (T790K) were overlapped at 95°C for 1 min, 65°C for 1 min, and 68°C for 4 min for 3 cycles, then extended and amplified with the same conditions for 25 cycles. NS3-A5990C (E490A) respective fragments were overlapped at 95°C for 30 s, 65°C for 30 s, and 68°C for 1.5 min, for 3 cycles, then extended and amplified for 25 more cycles under the same conditions. The overlapped fragments were purified *via* gel extraction for amplification purposes, the purified products were cloned into pGEM^®^-T Easy Vector using T4 DNA Ligase overnight at 4°C. Transformation of plasmid was done using Xl-1 blue competent cell, spread on LB agar plate containing 100 μg/ml ampicillin, and incubated at 37°C for 16 h. Blue-White screening was performed using a mixture of IPTG and x-gal. The plasmids were amplified in 3 ml LB containing 100 μg/ml ampicillin at 37°C for 16 h in a shaking incubator set to 225 rpm and were later cut with ScaI and KpnI for E-C1350A (T188T), BspEI and NheI for NS3-A5990C (E490A), ad NheI and NaeI for NS5-T7812G (G81G), NS5-T8919G (C450W), NS5-C9420A (A617A), and NS5-C9938A (T790K). The fragments were then introduced into the corresponding region of the pDENV2-1,006 plasmid that served as template, ligated with T4 ligase at 16°C, overnight. Transformation was done with One Shot™ Stbl-3™ competent cells, was spread on LB agar plates containing 35 μg/ml kanamycin and incubated at 29°C for 3 days. Single colonies were selected and were further cultured in LB broth containing 35 μg/ml kanamycin at 29°C for 36–48 h in a shaker incubator set to 225 rpm. All plasmids were purified using Real Genomics HiYield^™^ Plasmid Mini Kit (RBC BioScience) and QIAGEN Plasmid Midi Kit (QIAgen).

### Production of rg Viruses

All plasmids underwent endotoxin removal using MiraCLEAN^®^ Endotoxin Removal Kit, according to manufacturer’s protocol. Plasmids were transfected into BHK-21 cells (4 × 10^5^/well) in 6-well plates using PolyJet^™^ *in vitro* DNA Transfection Reagent (SignaGen) according to manufacturer’s instructions. pDENV2-1,006 possesses the EGFP sequence within its plasmid, therefore is able to emit fluorescence. Reverse genetics (rg) viruses were harvested 6–7 days post-transfection, or when cytopathic effect (CPE) and/or fluorescence were observed on more than 75% of cells. Viruses were sub-cultured in BHK-21 cells (passage 1, P1 virus). With the presence of the EGFP sequence, P1 viruses were sequenced to confirm the presence of the mutations and quantified *via* flow cytometry.

### Flow Cytometry

In flow cytometry, BHK-21 cells were seeded in 24-well plates (1 × 10^5^/well) and incubated for 24 h. Wells were infected with 50 μl of viral supernatant and made up to 100 μl per well. Plates were incubated at 37°C for 2 h, rocking every 15 min. Wells were then washed with PBS, refreshed with 1 ml of cell medium, and incubated for 2 days at 37°C. After the incubation period, the supernatant was aspirated, wells were washed with PBS, and 1× trypsin was added to detach and resuspend cells. Cells were then fixed with 4% paraformaldehyde (PFA) and incubated at room temperature for 30 min, after which the cells were centrifuged for 5 min at 750×*g*-force. Cells were washed and resuspended with 1 ml of PBS and were ready to be titrated *via* the flow cytometer. Viral titer in fluorescent forming units per milliliter (FFU/mL) was calculated as follows:


$$FFU/\mathrm{mL}=\frac{\mathrm{number of cells infected}\times \%\mathrm{EGFP positive cells}\;}{\mathrm{volume of virus used}}$$

### Elispot

Vero cells (2 × 10^4^/100 μl/well) were seeded in a 96-well plate and cultured at 37°C, 5% CO2 for 24 h. Cells were infected with 30 μl of ½ log serially diluted viruses. Virus adsorption was carried out for 1 h, after which 150 μl of overlay medium, containing EMEM virus medium and 1% methyl cellulose, were added into each well. Supernatant was aspirated 4 days post-infection, wells were washed twice with 180 μl of PBS, and then fixed with 100 μl of 80% methanol. Once fixed, immunostaining was performed. Blocking was done using 5% skimmed milk in PBS, cells were then stained with 100 μl/well of MAB8705, mouse anti-dengue complex monoclonal antibody (Millipore) and incubated for 2 h at 37°C on a shaker set to 30 rpm. Cells were washed 4 times with PBS-T (0.05% Tween 20 in PBS), stained with 100 μl/well of Goat anti-mouse IgG conjugate HRP (KPL (#5220–0460), LGC Seracare, Milford MA, United States of America), incubated at 37°C on a shaker for 1 h, then washed six times with PBS-T. TrueBlue Peroxidase Substrate (KPL (#5510–0030); LGC Seracare, Milford, MA, United States of America) was added to each well and incubated at room temperature for 20 min in the dark. The number of spots and photographs of each well was then taken using the ImmunoSpot system and softwares ImmunoSpot Image Acquisition and cell counting software (CTL-ImmunoSpot, Cleveland, OH, United States of America). The focus forming units per milliliter (FFU/mL) was then calculated.

### Virus Growth Kinetics

Baby hamster kidney cells (BHK-21), Vero, and Huh-7 cells (2 × 10^5^/ml) were seeded in 24-well plates and incubated for 24 h. The cells were infected with 100 μl of virus with a MOI of 0.01 in virus culture medium (BHK-21 and Vero: EMEM, 2% FBS, 2% P/S, 1× sodium pyruvate; Huh-7: DMEM, 2% FBS, 2% P/S). Viruses were collected at 0-, 1-, 2-, 3-, and 4-days post-infection. Collected virus samples were quantified to obtain viral titer.

### Thermal Sensitivity and Stability

For thermal sensitivity, viruses of 1 × 10^3^ FFU (MOI: 0.01) were used to infect BHK-21, Vero, and Huh-7 cells and were incubated at 35, 37, and 39°C. Viruses were collected 4 days post-infection. Collected virus samples were quantified by flow cytometry to measure the titer of viruses. For thermal stability, viruses of 5 × 10^5^ FFU in 150 μl viral culture medium were incubated at 29, 37, and 39°C using a thermocycler for 0, 30, 60, 90, and 120 min. The viruses were chilled on ice prior to and after the incubation period. Viruses were then quantified to measure the amount of virus that remained infectious.

### Statistical Analysis

Statistical analysis of LoFreq data was performed by Chi-square using SPSS. Haplotype data comparison analysis was performed using t-test in GraphPad Prism. All other data were analyzed using two-way analysis of variance (ANOVA). A value of *p* < 0.05 was defined as statistically significant. The data were analyzed by GraphPad Prism 5.

## Results

### Haplotype Analysis

**Figure 1:**
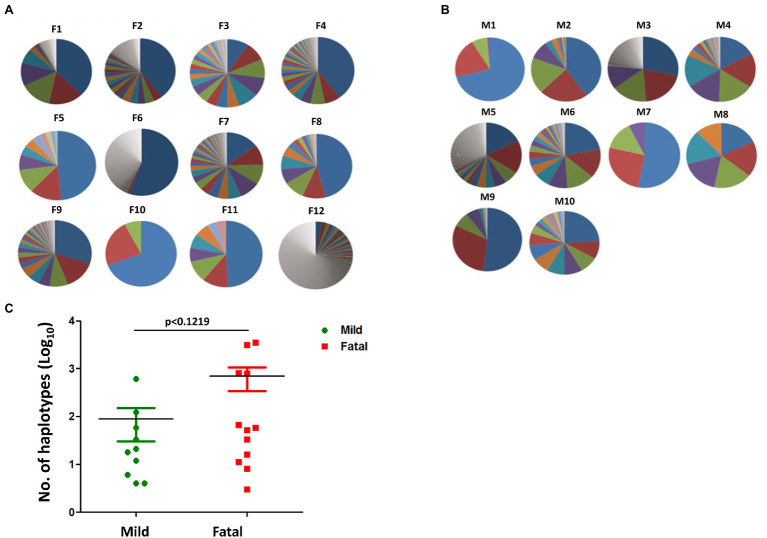
Haplotypes of mild and fatal cases. Proportion of haplotypes within each isolate in both **(A)** fatal and **(B)** mild cases. Each pie chart represents a single isolate. The different sections within the chart each represent a percentage of a haplotype, whereby the bigger the section the higher the percentage. The major haplotype is represented by the section with the highest percentage. Similar section color does not represent the same haplotype and does not represent similar sequence. Each section represents a single individual haplotype. **(C)** The number of haplotypes between mild (green) and fatal (red) cases. Each point represents a single isolate. Student’s *t*-test, *p* < 0.1219.

To determine whether there is any significant difference in DENV genome variations between mild and fatal cases during the 2015 outbreak, 22 clinical samples were analyzed. Among the 22 isolates were 10 from mild cases and 12 from fatal cases (Supplementary Table S1). Haplotypes are defined as the viral population that represents the quasispecies of the virus. These viral populations, although being derived from a parental virus, can differ from one another due to mutations or any other changes within its sequence. In the dataset provided from NGS, several thousands of reads were obtained on the viral genome. Each of these reads represents a single haplotype which makes up a percentage of the viral population. The haplotype with the largest percentage represents the major haplotype. In order to determine whether the number of haplotypes may have contributed to the disease severity, the total number of haplotypes per isolate was compared using GraphPad Prism. As shown in Figure 1, there was no visible difference between the number of haplotypes found in the fatal cases and those found in the mild cases. Both sets of isolates had relatively high number of haplotypes, as well as relatively low number of haplotypes. When comparing the total number of haplotypes between mild and fatal cases, the total average of the haplotypes from fatal cases was higher than that of the average total from mild cases (*t*-test, *p* value = 0.1219; Figure 1C). However, this alone cannot be used to determine disease severity since every isolate is different from the other. Therefore, it remains unclear whether the number of haplotypes had an impact on disease severity in the 2015 epidemic.

### Variation Analysis of Mild and Fatal Cases by Lofreq

Since a higher average total but no significant difference (*p* = 0.1219) in haplotypes was seen between mild and fatal case isolates, we further investigated the variations found in the haplotype population between mild and fatal cases. The NGS data, after being analyzed with QuasiRecomb, provided information on which nucleotide variation had the highest frequency at each position. Using data obtained from QuasiRecomb, variation at each position of each isolate was calculated for both mild cases and fatal cases. LoFreq, whilst it only provides information on selected positions that are unique, a total of 815 positions were obtained for further analysis (Supplementary Table S3). Majority were located in the NS region with the bulk of it being in the NS5 region (Supplementary Table S4). The variation at each position selected by LoFreq for each isolate was then determined. Those with a significant difference between mild and fatal cases were further determined using LoFreq: overall flow of sample preparation and analysis was shown in Supplementary Figure S1.

**Figure 2:**
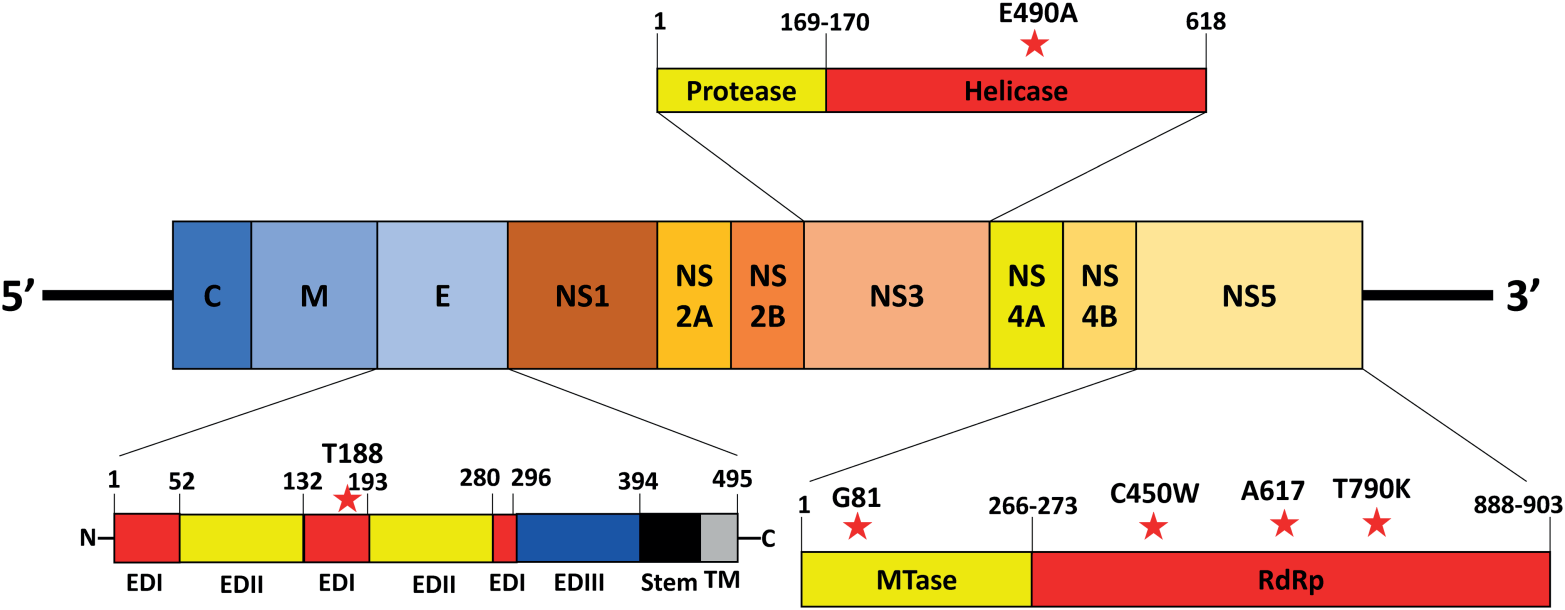
Location of nucleotide variations in virus genome. The location of the nucleotide variations within the DENV viral genome. The red stars represent the variations on the virus genome.

**Figure 3:**
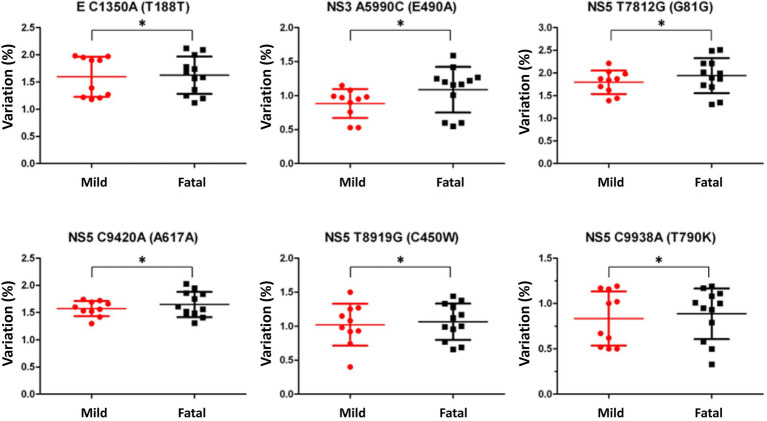
Nucleotide variation comparison in mild and fatal cases. Variation comparison of the selected positions between mild (red) and fatal (black) cases. The variations selected by LoFreq with a *p*-value <0.05 showed that fatal cases had higher variation percentage compared to mild cases. Statistical difference of variation percentages at each position between mild and fatal cases was calculated using Chi-square analysis ^*^p < 0.05.

The variant nucleotide is defined as the nucleotide at a single position having significance compared to wildtype. To investigate whether the variant nucleotides had a greater composition in either mild cases or fatal cases, the mean variation frequency of mild cases and that of fatal cases obtained from QuasiRecomb were further compared and analyzed. Based on the 815 unique positions selected from LoFreq, the DENV genome wildtype nucleotides were first determined for each isolate at each position, after which the variant nucleotide was determined. Using Chi-square analysis, when comparing the variant nucleotides of mild cases with those of fatal cases within the DENV genome, LoFreq resulted in 6 positions that were significantly different with value of ps < 0.05. Looking more closely at the 6 positions alone, a difference in variation on the positions was seen. The six positions, namely 1,350, 5,990, 7,812, 8,919, 9,420, and 9,938, were found in the E protein, NS3 and NS5, respectively (Table 1; Figure 2). With the substitution, both synonymous and non-synonymous mutations were observed. There was a total of three silent mutations, and the remaining three were non-synonymous mutations, which resulted in amino acid substitutions (Table 1). Additionally, the variation percentages in fatal cases were slightly higher than mild cases (Figure 3). Therefore, the variations may have an impact on disease severity.

**Table 1:** Mutations and amino acid substitutions at the six positions.

Protein (region)	Position (nt)	Amino acid (aa)	Wildtype nucleotide	Variant nucleotide	Change in aa
E (Domain I)	1,350	T188	C	A	No
NS3 (Helicase)	5,990	E490	A	C	E → A
NS5 (MTase)	7,812	G81	U	G	No
NS5 (RdRp)	8,919	C450	U	G	C → W
NS5 (RdRp)	9,420	A617	C	A	No
NS5 (RdRp)	9,938	T790	C	A	T → K

*MTase, Methyltransferase; RdRp, RNA-dependent RNA polymerase; No, no mutation (synonymous/silent mutation); nt, nucleotide; aa, amino acid. Location of positions from LoFreq with the viral genome’s nucleotide position number, viral protein amino acid number and amino acid, wildtype and variant nucleotide at the position, and any amino acid change which occurred when substituting wildtype nucleotide with the variant nucleotide. Variations were found to have a higher frequency in fatal cases than in mild cases*.

### Production of Reverse Genetics (rg) Viruses

To investigate the effects of the variant nucleotides in viruses, we substituted the wildtype nucleotide with the variant nucleotide. Reverse genetics virus system allows us to examine the effect of the genetic substitutions and rules out the effect of any other variations. The production of rg-viruses was confirmed by examination *via* a fluorescent microscope. Reverse genetics viruses of NS5-T7812G (G81G) and NS5-C9420A (A617A) were successfully produced (Supplementary Figure S2). The rg-viruses containing the remaining variations were unable to be obtained regardless of the numerous attempts. This may be due to the toxicity on *E. coli* during transformation and on BHK-21 cells during transfection into cells. We therefore focused on investigating the impact of the NS5-T7812G (G81G) and NS5-C9420A (A617A) substitutions on the viral properties in this study. The viral titers of the produced rg-viruses were determined using flow cytometry and mutant variants were confirmed by Sanger sequencing (Genomics).

### Replication Rate of Virus Variants rgNS5-T7812G and rgNS5-C9420A Were Better Than WT Virus

**Figure 4:**
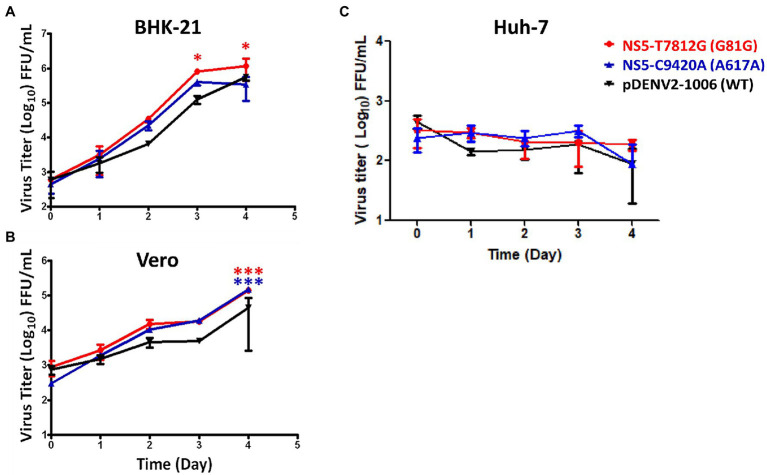
Multiple-step replication cycle of DENV variants and WT. BHK-21 **(A)**, Vero **(B)**, and Huh-7 **(C)** cells were infected with viruses containing the variants and WT (pDENV2-1,006 NS5-7812 T/9420C) virus at a MOI of 0.01. Virus titers were quantified at 0-, 1-, 2-, 3-, and 4-days post infection *via* flow cytometry and ELISpot. Titers were calculated for FFU/mL. The circle, triangle, and inverted triangle symbols represent NS5-T7812G (G81G), NS5-C9420A (A617A), and pDENV2-1,006 (WT- NS5-7812 T/9420C) viruses, respectively. Statistical difference comparing mutants with wildtype was calculated using two-way ANOVA. ^*^p < 0.05; ^***^p < 0.001.

We investigated the viral multiple-step replication cycle growth kinetics in BHK-21, Vero, and Huh-7 cells. Cells were infected by viruses with concentration of low multiplicity of infection (MOI 0.01). The cell lysates were collected daily for up to 4 days post-infection (dpi) and viral titers were determined. In BHK-21 cells, NS5-T7812G (G81G) had a significantly higher titer than WT viruses at 3 and 4 dpi (Figure 4A). In Vero cells, both NS5-T7812G (G81G) and NS5-C9420A (A617A) had significantly higher titers than WT virus at 4 dpi (Figure 4B). When quantifying infected cells in Huh-7, little to no fluorescence was detected, therefore we used ELISpot to determine the presence of viruses in the samples collected. Mutant and WT virus showed no difference in Huh-7 cells, and it appears as though viral replication did not increase among any of the viruses (Figure 4C). These results indicate that the virus variants had better replication rates than WT in both BHK-21 and Vero cells.

### Both rgNS5-T7812G and rgNS5-C9420A Were Temperature Sensitive

**Figure 5:**
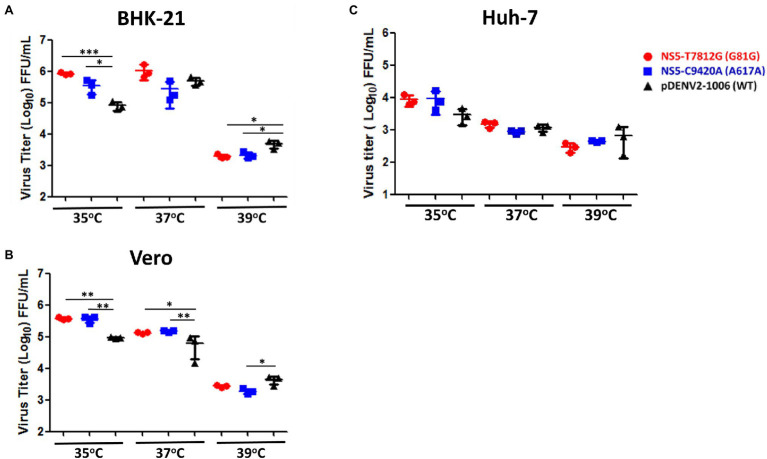
Thermal sensitivity of virus variants and WT virus. BHK-21 **(A)**, Vero **(B)**, and Huh-7 **(C)** cells were infected with virus variants and WT virus at a MOI of 0.01. Infected cells were incubated at three different temperatures (35°C, 37°C, 39°C) for 4 days. Virus titers were quantified *via* flow cytometry and ELISpot. Titers were calculated for FFU/mL. The circle, square and triangle symbols represent NS5-T7812G (G81G), NS5-C9420A (A617A), and pDENV2-1,006 (WT- NS5-7812 T/9420C) viruses, respectively. Asterisks (^*^) indicate a significant difference with WT. Statistical difference comparing virus variants with wildtype was calculated using two-way ANOVA. ^*^p < 0.05, ^**^p < 0.01, and ^***^p < 0.001.

Different incubation temperatures were used in order to determine the thermal sensitivity of the virus variants. BHK-21, Vero, and Huh-7 cells were infected by the rg-viruses and incubated at 37°C for 1 h for adsorption. The viruses were then allowed to grow in 35°C, 37°C, and 39°C for up to 4 dpi, and then quantified. In BHK-21 cells, both NS5-T7812G (G81G) and NS5-C9420A (A617A) had significantly higher titers than WT virus at 35°C, but significantly lower titers than WT virus at 39°C (Figure 5A). In Vero cells, at 35°C and 37°C, NS5-T7812G (G81G) and NS5-C9420A (A617A) both had significantly higher titers compared to WT virus. Contrastingly, at 39°C, both NS5-T7812G (G81G) and NS5-C9420A (A617A) had low titers but only rgNS5-C9420A (A617A) had a significantly lower titer than WT virus (Figure 5B). Note to mention, NS5-T7812G (G81G) and NS5-C9420A (A617A) both had lower titers compared to WT in BHK-21 and Vero cells at 39°C. In Huh-7 cells, there was no significant difference between WT virus and mutant viruses at 35, 37, and 39°C (Figure 5C). Additionally, higher titers of both NS5-T7812G (G81G) and NS5-C9420A (A617A) were seen at 35°C compared to 37°C, and 39°C in Huh-7 cells. These results indicate that the rg-viruses had higher titers and can still readily replicate at 35°C and 37°C in comparison to WT viruses but were unable to replicate at a high temperature of 39°C. Therefore, WT virus is more resistant to higher temperatures than variant viruses.

### Effect of Substitution on Virus Thermal Stability

**Figure 6:**
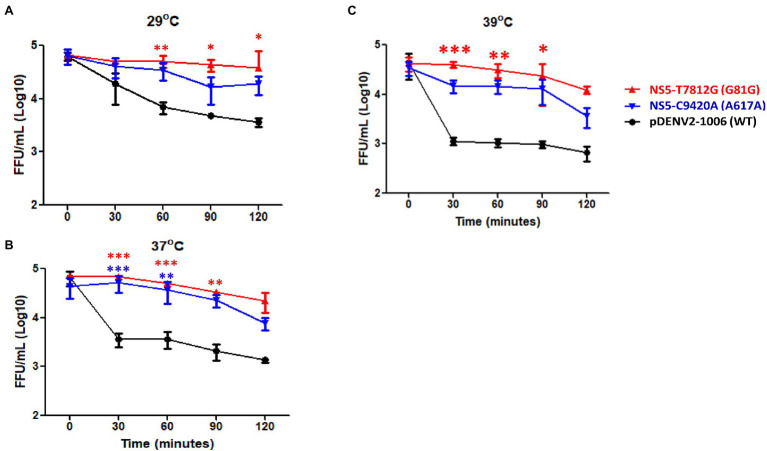
Thermal stability of virus variants and WT virus. Viruses were incubated at **(A)** ∆29°C, **(B)** ∆37°C and **(C)** ∆39°C for 0, 30, 60, 90, and 120 min using a thermocycler. Post incubation period, viral titers were quantified in Vero cells to determine the number of infectious viruses remaining. Titers were calculated for FFU/mL. The triangle, inverted triangle, and circle symbols represent NS5-T7812G (G81G), NS5-C9420A (A617A), and pDENV2-1,006 (WT-NS5-7812 T/9420C) variants, respectively. Statistical analysis comparing mutants with wildtype was calculated using one-way ANOVA. ^*^p < 0.05, ^**^p < 0.01, and ^***^p < 0.001.

We then investigated the stability of the variants to different temperatures. Using a thermocycler, 150 μl of virus (5 × 10^5^ FFU/mL) were incubated for 0, 30, 60, 90, and 120 min at 29, 37, and 39°C. These three temperatures were selected in order to represent the mosquito host, healthy human host, and an individual with fever, respectively. After treatment, the number of infectious particles remaining was quantified. At a temperature of 29°C, the number of remaining infectious particles was significantly higher at 60, 90, and 120 min in rgNS5-T7812G (G81G) compared to WT virus, while rgNS5-C9420A (A617A) showed no significant difference (Figure 6A). At 37°C, rgNS5-T7812G (G81G) and rgNS5-C9420A (A617A) had significantly higher titers than WT virus (Figure 6B). Interestingly, at 39°C, both variants had higher titers than WT virus but only rgNS5-T7812G (G81G) was significantly higher compared to WT virus (Figure 6C). These results indicate that the variant viruses NS5-T7812G (G81G) and NS5-C9420A (A617A) are able to remain infectious and stable at different temperatures for a longer duration compared to WT virus, especially at higher temperatures whereby the viral titers of WT virus drastically decreased over time.

## Discussion

Dengue virus serotype 2 caused one of the largest DENV outbreaks in 2015 that Taiwan had experienced. In the previous year 2014, a large outbreak DENV-1 had also occurred, resulting in 15,043 cases in Kaohsiung City. It was suggested that the 2014 outbreak may have been correlated to an underground pipeline explosion, leading to the increase in mosquito density (Wang et al., 2016b). The following year in 2015, this large outbreak of DENV-2 resulted in 22,777 cases in Tainan City and 19,784 cases in Kaohsiung City, with a total of 227 deaths (CDC, Taiwan). It was previously reported that the difference in severity during the 2015 outbreak may be due to previous infection with DENV-1 from 2014 and then later on being infected with DENV-2 from the 2015 outbreak (Wang et al., 2016a). Other factors such as age, pre-existing health conditions, and pre-existing dengue antibodies were also suggested to have been correlated with dengue severity during this outbreak (Kroschewski et al., 2008). Although this may be probable, there is also the possibility of other factors that may have played a role in this epidemic. Some of these factors include social and environmental factors such as importation and climate change (Wang et al., 2016b). In this study, we used NGS on DENV-2 viruses from both mild and fatal cases, isolated during the 2015 outbreak in southern Taiwan. Statistical analysis of the NGS data resulted in six variations that showed a significantly higher percentage of variations in fatal cases than in mild cases. These majority of the 6 variations were found in the nonstructural region of the DENV genome (Figure 2). We found that two variations, NS5-T7812G (G81G) and NS5-C9420A (A617A), resulted in silent mutations and were located in regions that play roles in viral replication. Replication analysis of rg-viruses containing the two variations showed that they had better replication rates at 35°C and 37°C compared to wildtype viruses in BHK-21 and Vero cells, but not in Huh-7 whereby limited replication was observed (Figure 4). NS5-T7812G (G81G) and NS5-C9420A (A617A) were also found to be temperature sensitive (Figure 5), however, were able to remain infectious for a longer duration, and were more thermally stable compared to wildtype viruses at 29, 37, and 39°C (Figure 6).

Next-generation sequencing has greatly increased the capacity at which we can examine variations of a virus. Not many studies have utilized NGS in determining the relationship between quasispecies and disease severity. However, in a study done by Vignuzzi et al., they had noticed that when mice are infected with a clone of poliovirus containing a diverse quasispecies, they developed diseases, while those infected with a genetically constrained population did not (Vignuzzi et al., 2006). Therefore, we tried to determine whether quasispecies played a role in disease severity in the 2015 DENV epidemic. Quasispecies consist of a virus population, and this virus population is made up of many haplotypes containing variations of the original virus. These haplotypes may differ from the original due to mutations in its sequence. Taking Vignuzzi’s work into consideration, the more haplotypes an isolate possesses, there may be a possibility that it is more lethal than an isolate with very little haplotypes (Vignuzzi et al., 2006). In our study we compared the number of haplotypes between mild cases and fatal cases (Figure 1). We found that both mild cases and fatal cases had isolates that possessed very few haplotypes as well as isolates with many haplotypes. When looking at the total number of haplotypes found in mild cases compared to fatal cases, the average of those found in fatal cases was greater than that of mild (Figure 1C). Although looking at the haplotype chart of each isolate individually may not provide any clues in the relationship between the number of haplotypes and disease severity, looking at the isolates all together shows some correlation to an increase in haplotypes leads to a more severe outcome (Figure 1C). Additionally, the number of samples used is not able to accurately convey a larger population. Further studies with an increased number of samples is required for analysis.

Huang et al. had observed that within an enterovirus A71 (EV-A71) viral population, two variations found at position 31 in the VP1 region resulted in different levels of virulence when infecting different regions of the body, indicating the role of selection pressure within a host (Huang et al., 2017). Parameswaran et al. had also demonstrated the effect of intrahost selection pressure on dengue virus (Parameswaran et al., 2017). Therefore, apart from the wildtype nucleotide that make up the viral populations, we hypothesized that the variant nucleotides may also affect viral properties which may play a role in the virulence of DENV. Using LoFreq, we were able to investigate these variant nucleotides that had a significant difference between mild and fatal cases. Since NGS along with bioinformatics programs can help us to identify significant variations that may have an impact on viral properties, both lethal and non-lethal variations can be resulted. In this study, some of the variations were not able to be successfully constructed in our reverse-genetics system due to either toxicity in *E. coli* during transformation or in BHK-21 cells during transfection. We hypothesize that the variation may be lethal, therefore the mutant virus was not able to be rescued. However, we were able to observe that the rg-viruses containing the variations NS5-T7812G (G81G) and NS5-C9420A (A617A) had a faster replication rate than WT in BHK-21 and Vero cells.

The substitution NS5-T7812G (G81G) was found to be located in the S-adenosyl-L-methionine (SAM) binding site in the NS5 methyltransferase (MTase) domain. SAM serves as a methyl donor in order for the methylation of guanine N7 and ribose 2′ -OH during viral cap formation to occur, therefore having an impact on viral replication. Kroschewski et al. demonstrated that single mutations in the SAM binding site either abolished or severely reduced both N7 and 2′-O MTase activities (Kroschewski et al., 2008), therefore influencing virus capping and in turn, virus production. However, in our study, rgNS5-T7812G resulted in a synonymous mutation. The amino acid was not changed but the codons used were different. This change in codon may have been beneficial rather than detrimental to virus replication, thus resulting in the variant virus having a faster replication rate and higher titer than WT. With respect to rgNS5-C9420A, the variant is located between motif B of the RdRp which is responsible for translocation and the setting of RNA into the RdRp tunnel for NTPs to bind to, and the RdRp active site (Liu et al., 2010; Van Slyke et al., 2012). NS5-C9420A (A617A) may impact translocation and NTP specificity, as well as the sliding of RNA in the RdRp tunnel (Kroschewski et al., 2008; Liu et al., 2010; Selisko et al., 2014; El Sahili and Lescar, 2017). Although the variation does not reside within the motif or in the active site, changes to this variation may influence activities upstream or downstream from it. There was also no change in amino acid with the variant (Table 1).

Although no change in protein structure was seen for E-C1350A (T188T), NS5-T7812G (G81G) and NS5-C9420A (A617A; data not shown), the change in codons resulted in a more preferred codon to be used. E-C1350A (T188T) resulted in a synonymous mutation whereby the threonine (T) at E protein position 188 (E-T188) changed from ACC to ACA. In the case of NS5-T7812G (G81G), the codon for glycine (G) at NS5-G81 changed from GGU to GGG which led to a change from the least preferred codon to the second highest used codon. For NS5-C9420A (A617A), the codon for alanine (A) at NS5-A617 changed from GCC to GCA resulting in a change from the second highest used codon to the most preferred codon (Supplementary Figure S3). Zhou et al. demonstrated the importance of codon usage and how each protein has a most and least preferred codon that is used during transcription (Zhou et al., 2016). The change in nucleotide caused a change in the codon used to one that is more preferred and may possibly be more advantageous to the virus itself. In all cases, the codon at the respective positions were optimized thus possibly having a positive effect on their respective protein functions and may also contribute to the variants being higher in fatal. Codon usage preference have also been found to have effects on the translation and initiation efficiency. Additionally, the use of preferred codons plays a role in regulating the transcriptional efficiency of DENV ORF in order to hijack the translational mechanisms of the hosts (Zhou et al., 2013). In the opposite whereby the least or lesser preferred codon is used, an attenuation may be seen (Manokaran et al., 2019; Tsai et al., 2019). Therefore, the variant nucleotides selected from quasispecies may have aided in the increase in virus replication.

In terms of thermal sensitivity of the variants, both variants were able to strive at temperatures of 35°C with a significantly higher titer than WT in both BHK-21 and Vero cells (Figure 5). At 37°C, both variants had significantly higher titers than WT in Vero cells but not in BHK-21 cells. This indicated that the variants at 35°C are considerably stable as compared to WT. However, at a temperature of 39°C, the viruses were unable to grow and resulted in the variant viruses having lower titers than WT. The stability of the viruses in different temperatures, as seen in Figure 6, may indicate the environmental impacts the viruses face when replicating in mosquitoes (29°C), healthy humans (37°C), and in an individual with fever (39°C). At lower temperatures found in the mosquito host (29°C), the virus tends to thrive for a longer duration, keeping its high titer and remaining infectious for a longer period of time. Both variant viruses were more stable than WT virus at a low temperature of 29°C. In the human host, both healthy (37°C) and with a fever (39°C), a gradual decrease in viral titer can be seen with the increase in temperatures. With higher temperatures, the virus cannot survive for long periods of time. However, NS5-T7812G (G81G) and NS5-C9420A (A617A) were more thermally stable than WT viruses at 37°C and 39°C, whereby WT virus had a drastic decrease of viral titer over time. With a change in codon to a more preferred sequence, the variant viruses may therefore thrive better in its mosquito host, amplifying in the mosquito before being passed on at higher titers to humans during infection. A summary of the results was shown in Table 2.

**Table 2:** Summary of results.

Virus	Cell type	Multiple-step growth curve	Thermal sensitivity (viral titer; 35°C/37°C/39°C)	Thermal stability (viral titer; 29°C/37°C/39°C)
rg-NS5-T7812G (G81G)	BHK-21	↑	↑/ns/↓	↑/ns/↑
	Vero	↑	↑/↑/ns	
	Huh-7	ns	ns/ns/ns	
rg-NS5-C9420A (A617A)	BHK-21	ns	↑/ns/↓	ns/ns/ns
	Vero	↑	↑/↑/↓	
	Huh-7	ns	ns/ns/ns	

*↑, significantly higher than WT;*

*↓, significantly lower than WT; and*

*ns, no significant difference compared to WT*.

In terms of virulence of dengue virus, two amino acids, E50A and G67A, located within the first transmembrane domain (TMD1) of the NS4A protein was shown to attenuate virus replication, decrease NS4A oligomerization and reduce stability (Lee et al., 2015). Additionally, Lin et al. had previously used the Taiwan DENV-22015 outbreak strain and successfully established a full transmission cycle using this virus (Lin et al., 2021). They demonstrated that the pre-membrane and envelope genes were key virulence determinants in the host and had high transmissibility of the Taiwan 2015 strain in mosquitoes. With the results of the investigation of these significant variant nucleotides that possessed silent mutations, further examination would be required to determine if any of the variations play a role in viral replication of DENV. Torres et al. previously analyzed the intrahost genetic diversity in patients of different clinical severity (Torres et al., 2021). Among the 385 non-synonymous single-nucleotide variants analyzed, 124 were highly detected among cases with warning signs and severe cases, however no single nucleotide variant was found to be responsible for the increase in severity (Torres et al., 2021). Therefore, with the lack of virulence determinants, increased importance must be placed in further identifying variants that contribute to viral replication.

A previous study has shown that a change in nucleotide A204G in the capsid coding region downstream from cHP structure restabilizes the structure, which is required for viral RNA synthesis, did not cause a change in protein, and resulted in a defect in viral replication (Clyde et al., 2008). In our study, majority of the variations between mild and fatal cases were found in the NS5 protein. Codon optimization within this region would result in improved protein expression, thus increasing the translational efficiency. This may partially explain why the variant nucleotides NS5-T7812G (G81G) and NS5-C9420A (A617A) had greater replication rates than wild types in BHK-21 and Vero cells. However, further investigation is required to further dissect the role of these variations in NS5 function. Additionally, a combination of mutations or a mixture of the variant viruses will be required for further investigation into the role of variants in quasispecies. The combination of viruses may bring insight into the cooperative effect of mutations on virus virulence compared to a single mutation.

In conclusion, the variations selected by LoFreq based on NGS sequencing had higher percentages in fatal cases. Although both variations resulted in silent mutations, the codons that resulted after substituting the wildtype with the variations had led to a more preferred codon being used. Replication rates of NS5-T7812G (G81G) and NS5-C9420A (A617A) were higher than WT in BHK-21 and Vero cells, are temperature sensitive, and are able to remain thermally stable and infectious at different temperatures compared to WT which had a drastic decline in titer at higher temperatures of 37°C and 39°C. Therefore, we have identified two variations thus far *via* quasispecies variation analyses that may contribute to the viral replication of DENV.

## Data Availability Statement

The data presented in the study are deposited in the BioProject repository (https://www.ncbi.nlm.nih.gov/bioproject), BioProject ID PRJNA814417, accession numbers SAMN26550919-SAMN26550940.

## Author Contributions

DC, S-WH, S-JH, and J-RW contributed to the conception and design of the study. DC, S-JH, and W-XC performed the experiments. DC, S-WH, and J-RW analyzed the data. DC and J-RW wrote the first draft of the manuscript. J-RW supervised the project and was in charge of funding and resource acquisition. S-WH, H-PT, JJHC, C-HC, and J-RW reviewed and edited the manuscript. All authors contributed to the article and approved the submitted version.

## Funding

J-RW received funding from the Ministry of Science and Technology (grant no. MOST109-2327-B-006-010) and from the National Health Research Institutes, Taiwan (grant nos. MR-110-GP-05 and MR-111-GP-06). The funders had no role in the study design, experimentation, data analysis, decision to publish, or the preparation of the manuscript.

## Conflict of Interest

The authors declare that the research was conducted in the absence of any commercial or financial relationships that could be construed as a potential conflict of interest.

## Publisher’s Note

All claims expressed in this article are solely those of the authors and do not necessarily represent those of their affiliated organizations, or those of the publisher, the editors and the reviewers. Any product that may be evaluated in this article, or claim that may be made by its manufacturer, is not guaranteed or endorsed by the publisher.

## Supplementary Material

The Supplementary Material for this article can be found online at: https://www.frontiersin.org/articles/10.3389/fmicb. 2022.894200/full#supplementary-material
