# Supplementary material for: Impact of Intrahost NS5 Nucleotide Variations on Dengue Virus Replication
Source: Front Microbiol. 2022 Jul 5;13:894200. doi: 10.3389/fmicb.2022.894200 (PMC9294511; doi:10.3389/fmicb.2022.894200)
Supplement: Supplementary file 1 [file Data_Sheet_1.zip › Supplementary Figures.PPTX]

## Slide 1
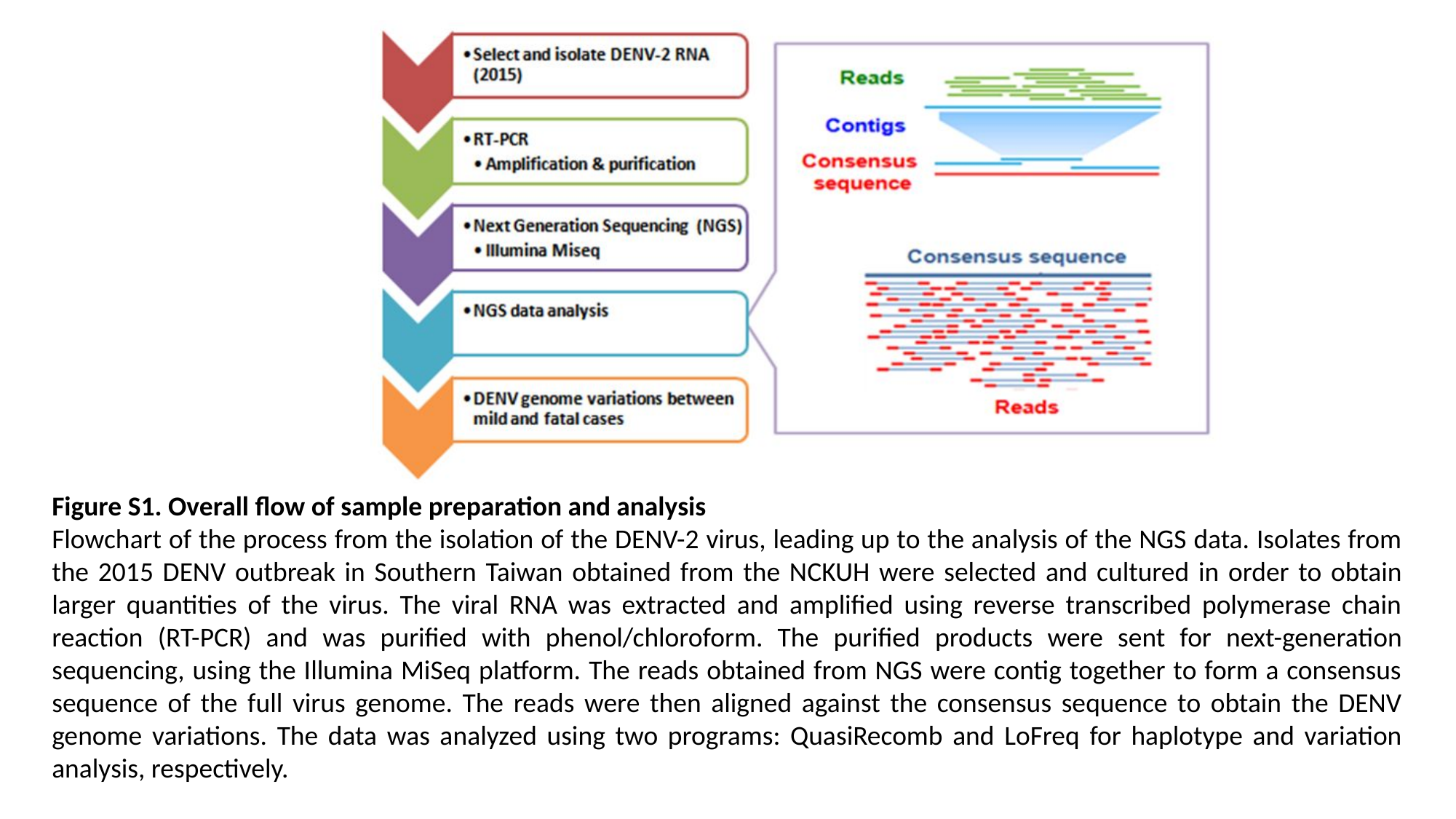

Figure S1. Overall flow of sample preparation and analysis
Flowchart of the process from the isolation of the DENV-2 virus, leading up to the analysis of the NGS data. Isolates from the 2015 DENV outbreak in Southern Taiwan obtained from the NCKUH were selected and cultured in order to obtain larger quantities of the virus. The viral RNA was extracted and amplified using reverse transcribed polymerase chain reaction (RT-PCR) and was purified with phenol/chloroform. The purified products were sent for next-generation sequencing, using the Illumina MiSeq platform. The reads obtained from NGS were contig together to form a consensus sequence of the full virus genome. The reads were then aligned against the consensus sequence to obtain the DENV genome variations. The data was analyzed using two programs: QuasiRecomb and LoFreq for haplotype and variation analysis, respectively.

## Slide 2
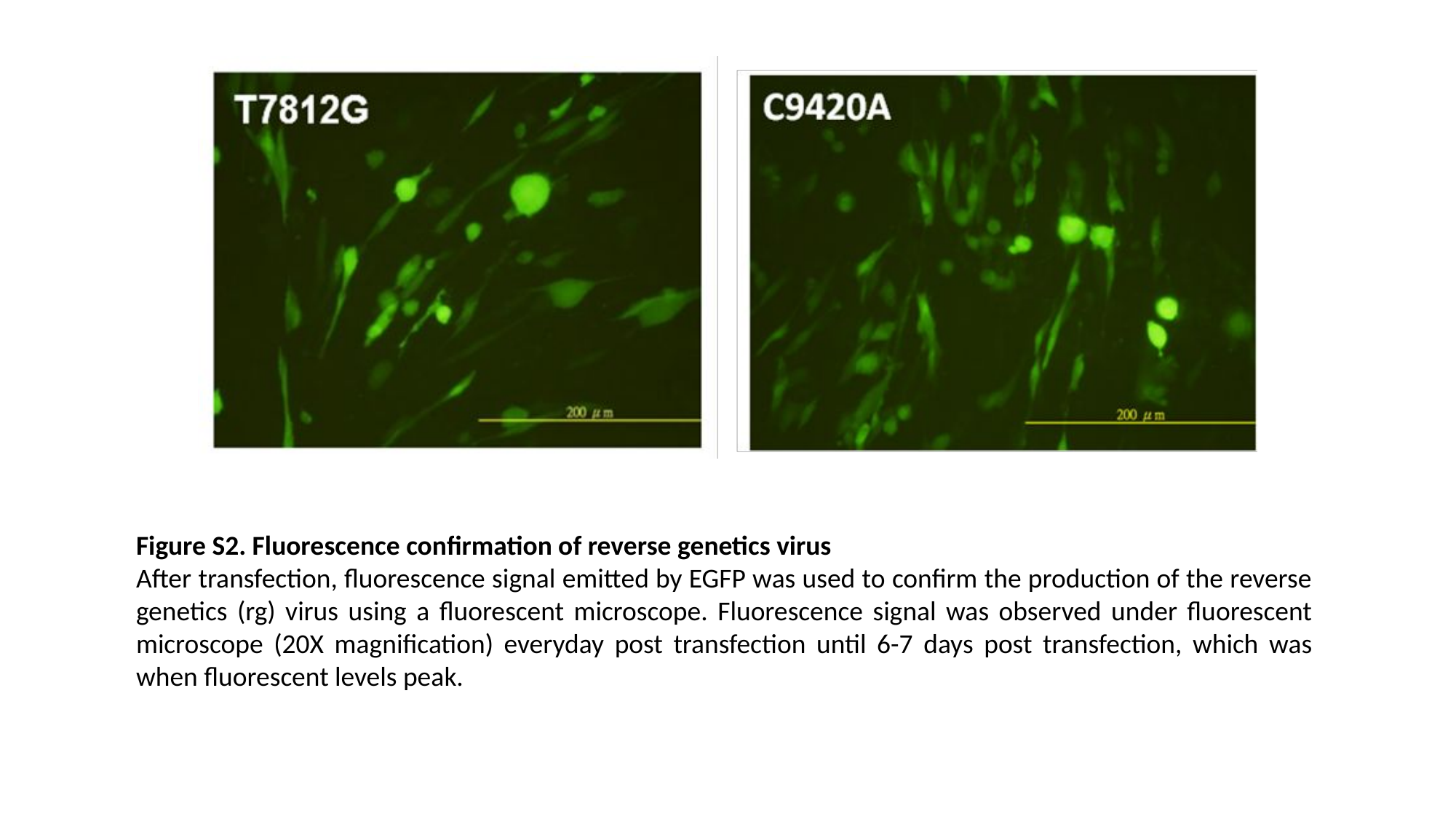

Figure S2. Fluorescence confirmation of reverse genetics virus
After transfection, fluorescence signal emitted by EGFP was used to confirm the production of the reverse genetics (rg) virus using a fluorescent microscope. Fluorescence signal was observed under fluorescent microscope (20X magnification) everyday post transfection until 6-7 days post transfection, which was when fluorescent levels peak.

## Slide 3
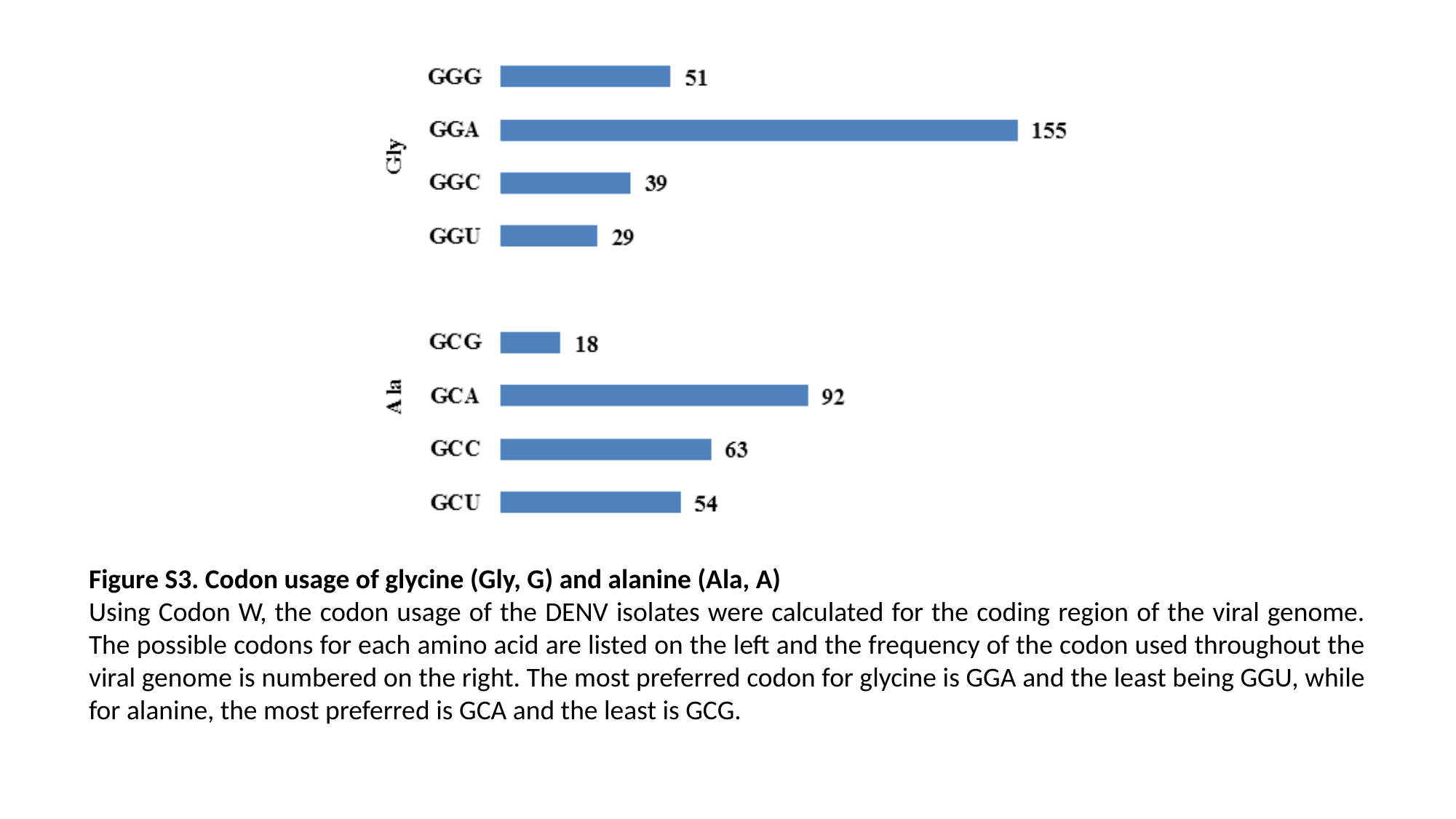

Figure S3. Codon usage of glycine (Gly, G) and alanine (Ala, A)
Using Codon W, the codon usage of the DENV isolates were calculated for the coding region of the viral genome. The possible codons for each amino acid are listed on the left and the frequency of the codon used throughout the viral genome is numbered on the right. The most preferred codon for glycine is GGA and the least being GGU, while for alanine, the most preferred is GCA and the least is GCG.
