# Supplementary material for: Impact of Intrahost NS5 Nucleotide Variations on Dengue Virus Replication
Source: Front Microbiol. 2022 Jul 5;13:894200. doi: 10.3389/fmicb.2022.894200 (PMC9294511; doi:10.3389/fmicb.2022.894200)
Supplement: Supplementary file 1 [file Data_Sheet_1.zip › Supplementary Tables.DOCX]

**Table S1. List of clinical isolates**

| **Mild Cases** | **Fatal Cases** |
| --- | --- |
| 15018925TW2015-M1 | 15017191TW2015-F1 |
| 15019638TW2015-M2 | 15018157TW2015-F2 |
| 15026672TW2015-M3 | 15018939TW2015-F3 |
| 15026786TW2015-M4 | 15019048TW2015-F4 |
| 15027362TW2015-M5 | 15019181TW2015-F5 |
| 15027632TW2015-M6 | 15020029TW2015-F6 |
| 15029493TW2015-M7 | 15021719TW2015-F7 |
| 15031689TW2015-M8 | 15022279TW2015-F8 |
| 15031711TW2015-M9 | 15025563TW2015-F9 |
| 15033612TW2015-M10 | 15025816TW2015-F10 |
|  | 15025975TW2015-F11 |
|  | 15027688TW2015-F12 |

Mild and fatal clinical isolates obtained from the National Cheng Kung University Hospital (NCKUH), Tainan, Taiwan.

**Table S2. DENV Primers**

| **Primer Name** | **Sequence (5' to 3')** |
| --- | --- |
| **DENV Full Genome Amplification Primers** | |
| D2F | AGT TGT TAG TCT ACG TGG ACC G |
| D2Rv3 | CCA GTA TTA TTG AAG CTG CTA TCC A |
| D2Fw4 | AAT CAC AGA RAT GGG TAG GCT |
| D2R | TGG CGT TCT GTG CCT GGA ATG AT |
| **DENV Site-Directed Mutagenesis Forward Primers** | |
| pDV-ScaI_F | GCC ACC CTA AGG AAG TAC TGT ATA GAG GCA AAG C |
| pDV-C1350A_F | ACC AGA AAA CTT GGA ATA CAC AAT TGT GAT AAC ACC TC |
| pDV-BspEI_F | ACG TTC GTG GAA TTC CGG ACA TGA ATG |
| pDV-A5990C_F | GCA CAC TGG AAA GCA GCT AAA ATG CTC CTA G |
| pDV-NheI_F | CTG CAT AAT CAC GGC TAG CAT CCT ATG |
| pDV-T7812G_F | TGG ACC TCG GGT GTG GCA GAG GA |
| pDV-T8919G_F | GAA GGA AAG TGT GAA ACA TGG GTG TAC AAC ATG |
| pDV-C9420A_F | TCA CCA ATA TGG AAG CAC AAC TAA TCA GAC AGA TG |
| pDV-C9938A_F | ATT GGG TTC CAA AAA GTC GAA CAA CCT GGT |
| **DENV Site-Directed Mutagenesis Reverse Primers** | |
| pDV-C1350A_R | GAG GTG TTA TCA CAA TTG TGT ATT CCA AGT TTT CTG GT |
| pDV-KpnI_R | TCA CTT CCC ACA GGT ACC ATG CTG CTG |
| pDV-A5990C_R | CTA GGA GCA TTT TAG CTG CTT TCC AGT GTG C |
| pDV-NheI_R | CAT AGG ATG CTA GCC GTG ATT ATG CAG |
| pDV-T7812G_R | TCC TCT GCC ACA CCC GAG GTC CA |
| pDV-C9420A_R | CAT CTG TCT GAT TAG TTG TGC TTC CAT ATT GGT GA |
| pDV-C9938A_R | ACC AGG TTG TTC GAC TTT TTG GAA CCC AAT |
| pDV-NaeI_R | ATC CAG CCG GCG TCC CG |

**Table S3. Number of variations with significant difference selected from LoFreq**

| **Number of positions** | | |
| --- | --- | --- |
| **Total** | 815 |  |
| p<0.05 | 6 |  |
| p<0.01 | 0 |  |
| p<0.001 | 0 |  |

NGS data analyzed by LoFreq was used to calculate the statistical difference between the variant nucleotides of mild and fatal cases. Positions with statistically significant difference were investigated further. Statistically significant differences were calculated using Fisher’s exact test.

**Table S4. Composition of the total number of positions selected by LoFreq**

| **Position** | **Number of variations** |
| --- | --- |
| 5'-UTR | 1 |
| C | 15 |
| M | 57 |
| E | 150 |
| NS1 | 64 |
| NS2A | 14 |
| NS2B | 9 |
| NS3 | 90 |
| NS4A | 13 |
| 2K | 3 |
| NS4B | 45 |
| NS5 | 344 |
| 3'-UTR | 10 |
| Total | 815 |

The number of variations found that were selected by LoFreq. The highlighted row represents the protein that had the largest number of variations.
